# Comparison of the Utility of Single-Ear and Binaural Auscultations: A Diagnostic Accuracy Study

**DOI:** 10.7759/cureus.88673

**Published:** 2025-07-24

**Authors:** Masafumi Shimoda, Jumpei Aso, Yoshiaki Tanaka, Megumi Hirano, Kozo Morimoto, Kozo Yoshimori, Haruyuki Ishii, Ken Ohta, Takeshi Saraya

**Affiliations:** 1 Respiratory Disease Center, Fukujuji Hospital, Kiyose, JPN; 2 Department of Respiratory Medicine, Kyorin University Faculty of Medicine, Mitaka, JPN

**Keywords:** auscultation, binaural, monaural, respiratory sounds, single ear, stethoscope

## Abstract

Introduction: Single-ear (monaural) auscultation is occasionally used in lung examinations. In this method, a binaural stethoscope is placed in one ear, leaving the other ear free for listening to other sounds. To enhance the efficiency of medical examinations, interviews, and auscultation are sometimes performed simultaneously using single-ear auscultation. However, there are no studies reporting the accuracy of single-ear auscultation for lung examinations. Therefore, this study aimed to compare the utility of single-ear and binaural auscultations.

Materials and methods: We prospectively collected data from 35 respiratory physicians at Fukujuji Hospital and Kyorin University Faculty of Medicine from April 2024 to June 2024. Data on audible minimal volume, points earned from auscultation questions, and self-reported confidence levels for answers to auscultation questions were compared between single-ear and binaural auscultations in both quiet and noisy environments. Furthermore, participants were divided into those with a respiratory specialist qualification (specialist group) and those without the qualification (nonspecialist group), and subanalysis was performed between these two groups.

Results: Single-ear auscultation resulted in fewer earned points than binaural auscultation in both quiet environments (median 12 points (range 7-15) vs. 13 points (9-16), *p*<0.001) and noisy environments (median 12 points (range 5-16) vs. 13 points (8-16), *p*=0.008). The average self-reported confidence levels were also lower for single-ear auscultation (quiet environment: *p*<0.001; noisy environment: *p*<0.001). The average audible minimal volume was not different between the single-ear and binaural auscultations (quiet environment: *p*=0.814; noisy environment: *p*=0.051). Twenty-three participants (65.7%) scored 1 or more points higher during binaural auscultation than during single-ear auscultation in both environments. There was no difference between the respiratory specialists and nonspecialists in the number of participants exhibiting advantages during binaural auscultation or in the difference in average audible minimal volume, earned points from auscultation questions, or average self-reported confidence levels for answers to auscultation questions between single-ear and binaural auscultations.

Conclusion: The utility of single-ear auscultation for lung auscultation was less than that of binaural auscultation. Physicians should use a binaural stethoscope correctly and listen with both ears.

## Introduction

Auscultation of the lungs is an essential part of the physical examination, providing valuable diagnostic information for various pulmonary diseases [1]. Some physicians utilize single-ear (monaural) auscultation [2,3], in which a binaural stethoscope is placed in one ear while the other ear is used to listen to other sounds [2,3]. For anesthesiologists, monaural auscultation allows communication with the surgeon and attention to other theatre personnel, suction, and any other monitor or alarm sounds during auscultation [2]. Furthermore, this technique is also used during abdominal examinations, where its efficiency allows for concurrent medical interviews, given the relatively limited diagnostic value of abdominal auscultation, which is generally confined to bowel sounds, bruits, and friction rubs [1,3,4]. In contrast, assessing the lungs may be more complex than performing an abdominal examination, and lung auscultation can indicate the presence of various pulmonary and bronchial diseases [1]. Single-ear auscultation is also occasionally used for lung auscultation; however, there are no studies reporting whether the accuracy of single-ear auscultation matches that of binaural auscultation, and it is uncertain whether single-ear auscultation is suitable for clinical practice. Therefore, this study aimed to compare the utility of single-ear and binaural auscultations.

## Materials and methods

Study design and setting

We prospectively collected data from 35 participants who were respiratory physicians at Fukujuji Hospital, Kiyose, and Kyorin University Faculty of Medicine, Mitaka, Japan, from April 2024 to June 2024. The exclusion criteria included participants with hearing impairments and those who did not complete the study protocol, as described in a later section. None of the participants had prior experience with single-ear auscultation, and all were unfamiliar with this technique before the study. Participants answered auscultation questions in both quiet (less than 40 decibel (dB)) and noisy (50 dB or more and less than 60 dB) environments. Data on audible minimal volume, points earned from auscultation questions, and self-reported confidence levels for answers to auscultation questions for single-ear and binaural auscultations were collected in both environments. These data were compared between single-ear and binaural auscultations performed with a binaural stethoscope in each environment. Furthermore, participants were divided into two groups based on their qualifications. Those with a respiratory specialist qualification were placed in the specialist group, while those without this qualification were placed in the nonspecialist group. Subanalysis was then performed between these two distinct groups. The study was approved by the Institutional Review Boards of Kyorin University Faculty of Medicine (Approval number: 2367, dated May 9, 2024) and Fukujuji Hospital (Approval number: 24003, dated May 24, 2024) and all participants provided written informed consent. The decisions made by this board were based on and in accordance with the Declaration of Helsinki.

Single-ear and binaural auscultations

Binaural auscultation was conducted with participants inserting the stethoscope’s ear tips into both ears, as is typical in standard auscultation. Single-ear auscultation involved inserting the ear tips into only one ear, leaving the other ear free; participants could choose which ear to leave free. The device used was a binaural stethoscope from Hi-Stetho (Taito Corporation, Tokyo, Japan), which was connected to a computer. Each participant used the same ear throughout the study when performing single-ear auscultation.

Quiet and noisy environments

We evaluated environmental sounds using a noise metre (Digital Sound Level Meter WT85, Shenzhen Wintact Electronics Co., Ltd., Shenzhen, Guangdong, China). A quiet environment was defined as less than 40 dB, as recommended for nighttime [5]. The noisy environment was adjusted between 50 dB and 60 dB by playing a television. Generally, few people are moderately annoyed below 50 dB, and guidelines for community noise recommend that outdoor sound levels should not exceed 50 dB during the daytime [5].

Evaluation of audible minimal volume, earned points from auscultation questions, and self-reported confidence levels for answers to auscultation questions

Audible minimal volume, earned points from auscultation questions, and self-reported confidence levels for answers to auscultation questions for single-ear and binaural auscultations were evaluated. Participants took the auscultation test twice, with an interval of at least seven days between the first and second tests to minimize recall bias. Answers were not disclosed after the first test. Binaural auscultation was performed first during the first test, and single-ear auscultation was performed first during the second test. Participants listened to a normal respiratory sound as an example before starting the tests. No participants had been diagnosed with hearing loss during health check-ups.

First, participants listened to a normal sample, and the audible minimal volume was recorded as the volume at which participants could hear the sounds while the volume was increased by 1% every second from zero on a computer.

Next, participants listened to respiratory sounds and then answered questions based on what they heard. In the first test, eight questions were asked in both quiet and noisy environments. In the second test, the same questions were asked in a different order (Table 1).

**Table 1:** Respiratory sound lists for listening by participants. Eight questions were asked in both quiet and noisy environments in the first test. In the second test, the same questions were asked in a different order. For details regarding Q1 to Q8 and N1 to N8, please refer to Table 2.

Test	Auscultation questions	
	In quiet environment	In noisy environment
First test		
Binaural auscultation	Q1	N1
	Q2	N2
	Q3	N3
	Q4	N4
Single-ear auscultation	Q5	N5
	Q6	N6
	Q7	N7
	Q8	N8
Second test		
Single-ear auscultation	Q3	N1
	Q1	N4
	Q2	N3
	Q4	N2
Binaural auscultation	Q8	N7
	Q5	N6
	Q7	N8
	Q6	N5

Each sound was listened to twice for 15 seconds each time. Participants listened to the same respiratory sounds for both single-ear and binaural auscultations during the combined first and second tests. Different respiratory sounds were used for quiet and noisy environments (Table 2).

**Table 2:** Respiratory sounds collected from patients and healthy volunteers.

Respiratory sound	Sample	Diagnosis of patients
Normal sound	Q4	No disease
	Q6	
	N1	
	N7	
Wheezing	Q1	Bronchial asthma
	N4	
Coarse crackles	Q2	Pneumonia
	Q5	
	N3	
	N5	
Fine crackles	Q3	Interstitial lung disease
	Q8	
	N2	
	N8	
Coarse crackles and wheezing	Q7	Pneumonia with bronchiectasis
	N6	

Participants answered a total of eight questions across the first and second tests in both the quiet and noisy settings. Each question was assigned two points, for a total of 16 points. The answer options included normal sounds, wheezing, coarse crackles, fine crackles, crackles (unknown whether coarse or fine), and difficult to judge (multiple answers were allowed). If participants provided only one answer when two were correct, or vice versa, and if participants answered “crackles” for coarse or fine crackles, one point was awarded. During the auscultation tests, a single supervisor was present for all sessions to ensure consistency. Participants recorded their answers on a standardized answer sheet. Aside from instructions on the test number and switching between single-ear and binaural auscultation, no additional communication took place during the test. After completion, the answer sheets were collected and scored by the same supervisor according to predefined criteria. The calculated scores were considered the earned points from the auscultation questions. The self-reported confidence levels of participants’ answers were also evaluated. The average of these measurements and self-reported confidence levels was calculated. When the number of points earned from auscultation questions during binaural auscultation exceeded that during single-ear auscultation by 1 point or more, we defined this as an advantage of binaural auscultation.

Respiratory sounds and the normal sample were reproduced using Eko: Digital Stethoscope + ECG application (Eko Health, Inc., Emeryville, CA, USA) on a computer. The sound volume for auscultation questions and the audible minimal volume were set to 30% on a computer, which closely mimicked the sound levels of real auscultations. The sound volume settings of the Eko: Digital Stethoscope + ECG application were left at their default settings. Only one computer was used for the sound test in the study.

Collection of respiratory sounds

Respiratory sounds for the auscultation test in this study were collected from 12 patients and five volunteers with no disease at Fukujuji Hospital. Auscultation sounds were recorded using a Littmann® CORE Digital Stethoscope (3M Company, St. Paul, MN, USA). All patients and volunteers provided written informed consent. The collected respiratory sounds included five normal sounds, two wheezing sounds from bronchial asthma, four coarse crackles from pneumonia, four fine crackles from interstitial lung disease, and two coarse crackles with wheezing from pneumonia with bronchiectasis (Table 2). The accuracy of all respiratory sounds was confirmed by two respiratory physicians with respiratory specialist qualifications.

Statistical methods

All the data were analysed and processed using EZR (Easy R), version 1.53 [6]. The Wilcoxon signed-rank test was used to compare data between single-ear and binaural auscultations. The Mann-Whitney U test and Fisher's exact test were used to compare data between the specialist and nonspecialist groups, as well as by sex and by the use of the left or right ear for single-ear auscultation. The interpretation of Spearman’s correlation was based on the Dancey & Reidy criteria as follows: perfect (r=1 or -1), strong (1>r≥0.7 or -1>r≥-0.7), moderate (0.7>r≥0.4 or -0.7>r≥-0.4), weak (0.4>r≥0.1 or -0.4>r≥-0.1), or zero (r=0) [7]. The level of statistical signiﬁcance was set at p=0.05 (2-tailed).

## Results

The baseline characteristics of the study subjects are shown in Table 3.

**Table 3:** Baseline characteristics of the study subjects.

Variable	n=35
Age, median (range), years	35 (26-44)
Sex (male/female)	27/8
Duration since becoming a doctor, median (range), years	8 (3-22)
Respiratory specialist qualification, n (%)	14 (40.0)
Free ear during single-ear auscultation (right/left)	14/21

In both quiet and noisy environments, there was no difference in the average audible minimal volume between single-ear and binaural auscultations (quiet environment: median 11.5% (range 7.0-24.0) vs. 11.0% (5.5-19.5), *p*=0.814; noisy environment: median 15.0% (range 5.5-27.0) vs. 14.5% (5.5-36.0), *p*=0.051) (Table 4). Individuals reported lower average confidence levels for answers to auscultation questions during single-ear auscultation than during binaural auscultation (quiet environment: median 60.0% (range 30.0-80.0) vs. 70.0% (range 38.8-92.5), *p*<0.001; noisy environment: median 55.0% (range 26.3-78.8) vs. 65.0% (range 38.8-88.8), *p*<0.001).

**Table 4:** Comparisons between single-ear and binaural auscultations. The Wilcoxon signed-rank test was used to compare data between single-ear and binaural auscultations.

Parameter	Single-ear auscultation	Binaural auscultation	W-value	p value
Quiet environment				
Average audible minimal volume, median (range), %	11.5 (7.0-24.0)	11.0 (5.5-19.5)	261	0.814
Average self-reported confidence levels for answers to auscultation questions, median (range), %	60.0 (30.0-80.0)	70.0 (38.8-92.5)	17.5	<0.001
Noisy environment				
Average audible minimal volume, median (range), %	15.0 (5.5-27.0)	14.5 (5.5-36)	390	0.051
Average self-reported confidence level, median (range), %	55.0 (26.3-78.8)	65.0 (38.8-88.8)	41	<0.001

**Figure 1:**
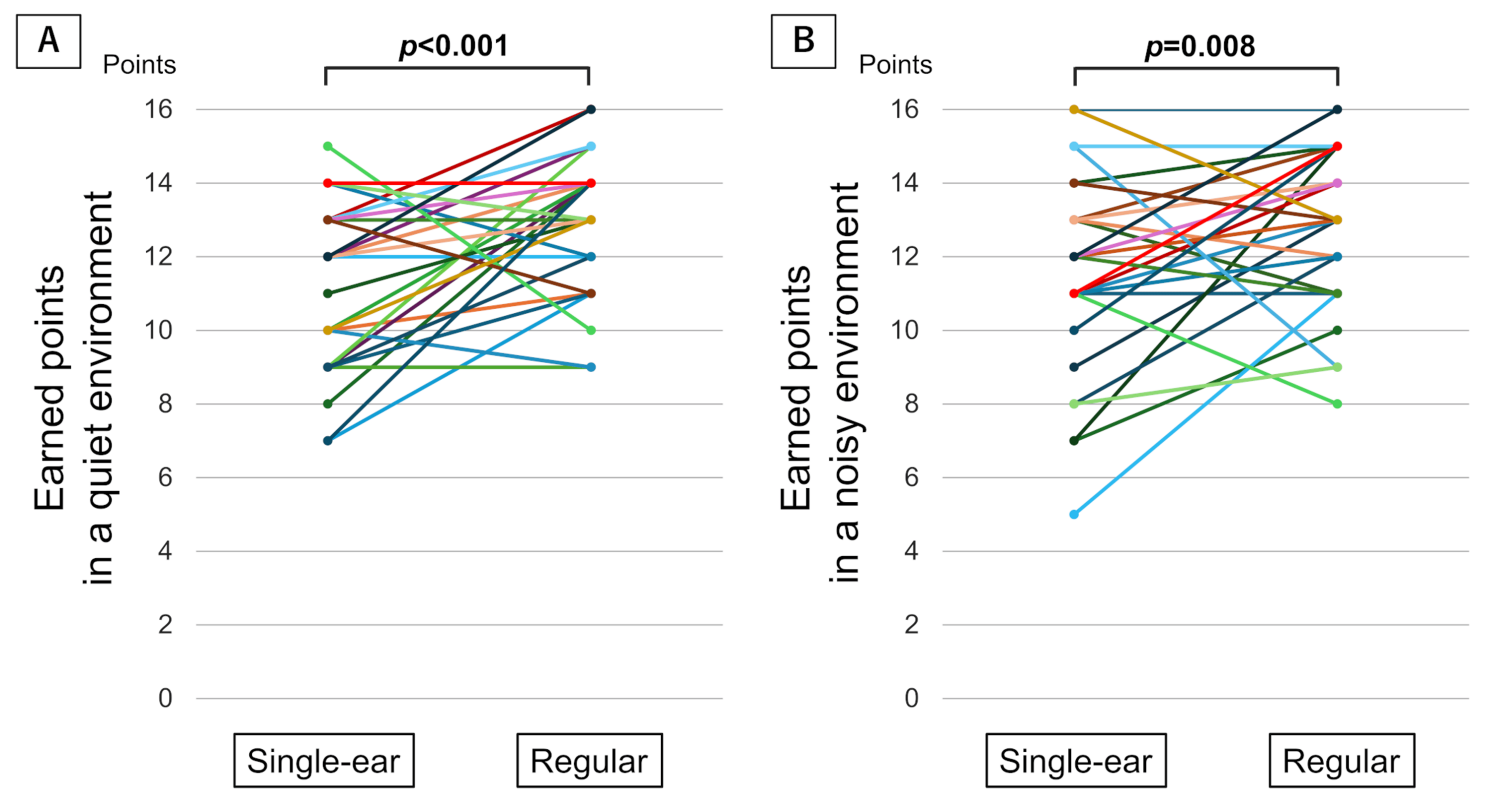
Comparisons of earned points from auscultation questions between single-ear and binaural auscultations. Each colored line represents an individual participant (n = 35). Note that some data points (circles) may overlap because multiple participants had identical values at those positions. A: Single-ear auscultations yielded fewer earned points than did binaural auscultations in a quiet environment (median 12 points (range 7-15) vs. 13 points (9-16), *p*<0.001). B: Single-ear auscultations yielded fewer earned points than did binaural auscultations in a noisy environment (median 12 points (range 5-16) vs. 13 points (8-16), *p*=0.008).

Comparisons of the earned points from the auscultation questions between the single-ear and binaural auscultations are shown in Figure 1. Single-ear auscultation resulted in fewer earned points than binaural auscultation in a quiet environment (median 12 points (range 7-15) vs. 13 points (9-16), *p*<0.001) and in a noisy environment (median 12 points (range 5-16) vs. 13 points (8-16), *p*=0.008). There were 23 (65.7%) participants who exhibited advantages with binaural auscultation in both quiet and noisy environments. Number of participants with advantages in binaural auscultation was higher in coarse crackles (n=22, 63%) compared to normal sound (n=7, 20%, *p*=0.045) and wheezing (n=5, 14%, *p*=0.048) in quiet environment, and was higher in combined coarse crackles and wheezing (n=16, 63%) compared to wheezing (n=3, 9%, *p*=0.039) in noisy environment, using comparative testing with Bonferroni correction.

Self-reported confidence levels for answers to auscultation questions had a linear relationship with earned points from auscultation questions. In single-ear auscultation, there was a moderate correlation between confidence levels and earned points in both quiet (r=0.498, *p*=0.002) and noisy (r=0.587, *p*=0.002) environments. In binaural auscultation, there was a weak correlation in both quiet (r=0.336, *p*=0.049) and noisy (r=0.338, *p*=0.047) environments.

Participants were divided into the specialist group (14 participants) and the nonspecialist group (21 participants) (Table 5). Regardless of the environment type (quiet or noisy), there was no difference between the specialist group and nonspecialist group in the number of participants exhibiting advantages during binaural auscultation or in the difference in average audible minimal volume, earned points from auscultation questions, or average self-reported confidence levels for answers to auscultation questions between single-ear and binaural auscultations.

**Table 5:** Comparisons between participants with and without a respiratory specialist qualification. The Mann–Whitney U test and Fisher's exact test were used to compare data between the specialist and nonspecialist groups.

Parameter	Specialist group (n=14)	Nonspecialist group (n=21)	U-value	p value
Quiet environment				
Number of participants with advantages in binaural auscultation, n (%)	8 (57.1)	15 (71.4)	-	0.477
Difference between binaural and single-ear auscultations				
For average audible minimal volume, median (range), %	-0.5 (-16.0-3.0)	1.0 (-6.0-5.5)	101	0.121
For earned points from auscultation questions, median (range), points	1 (-5-7)	2 (-2-5)	136	0.722
For average self-reported confidence levels for answers to auscultation questions, median (range), %	8.8 (1.3-37.5)	7.5 (-8.1-31.3)	159	0.698
Noisy environment				
Number of participants with advantages in binaural auscultation, n (%)	9 (64.3)	14 (66.7)	-	>0.999
Difference between binaural and single-ear auscultations				
For average audible minimal volume, median (range), %	-1.0 (-6.0-18.0)	-1.0 (-7.0-4.5)	157	0.748
For earned points from auscultation questions, median (range), points	1 (-6-5)	1 (-2-8)	139	0.785
For average self-reported confidence levels for answers to auscultation questions, median (range), %	10.6 (-2.5-26.3)	8.8 (-11.3-41.3)	161	0.649

Furthermore, no significant differences were observed between males and females in the difference in average audible minimal volume between binaural and single-ear auscultations (quiet environment: p=0.937; noisy environment: *p*=0.355), the difference in earned points from auscultation questions (quiet environment: *p*=0.417; noisy environment: *p*=0.283), and the difference in average self-reported confidence levels (quiet environment: *p*=0.397; noisy environment: *p*=0.062). Similary, age was also not significantly correlated with average audible minimal volume between binaural and single-ear auscultations (quiet environment: r=-0.199, p=0.252; noisy environment: r=0.126, p=0.472), the difference in earned points from auscultation questions (quiet environment: r=0.659, p=0.659; noisy environment: r=0.131, p=0.454), and the difference in average self-reported confidence levels (quiet environment: r=0.131, p=0.454; noisy environment: r=-0.07, *p*=0.699). There was no significant difference in the difference in earned points from auscultation questions regarding whether the left or right ear was used for single-ear auscultation (quiet environment: *p*=0.839; noisy environment: *p*=0.633). 

No adverse events were observed by any participants during the study.

## Discussion

This study demonstrated that the utility of single-ear auscultation for lung auscultation is less than that of binaural auscultation. The lower number of points earned from auscultation questions during single-ear auscultation than during binaural auscultation indicated that physicians could misdiagnose patients using single-ear auscultation. This result was observed in both quiet and noisy environments. When comparing individual auscultatory sounds, wheezing was relatively easy to identify, and many participants showed no difference in performance between single-ear and binaural auscultation. In contrast, coarse crackles tended to be more difficult to distinguish with single-ear auscultation. Based on these findings, single-ear auscultation may be inadequate for detecting respiratory sounds associated with pneumonia or interstitial lung disease. Additionally, self-reported confidence levels for answers to auscultation questions were lower while performing single-ear auscultation. The correct answer rate for auscultation questions and self-confidence levels for these answers were proportional. Furthermore, there was no difference in the nonutility of single-ear auscultation between respiratory specialists and nonspecialists. Accordingly, single-ear auscultation for lung auscultation might lead to decreased diagnostic accuracy regardless of the environment or clinical experience. 

Generally, binaural listening is superior to monaural listening in everyday listening situations [8], where binaural listening can make sounds appear about 3-6 dB louder than monaural listening [9,10]. Additionally, when a sound signal and noise are sent to only one ear, individuals may struggle to identify the sound signal (binaural squelch) [10]. However, in this study, there was no difference in the average audible minimal volume. This might be associated with the phenomenon where the listener can filter out background noise and focus solely on the target speaker (the cocktail party effect) [11]. Since participants concentrated on listening to the auscultation sounds, the cocktail party effect might have been applied. Nevertheless, auscultation accuracy was higher with binaural auscultation compared to single-ear auscultation. Therefore, while it is possible to hear sounds with single-ear auscultation, the diagnostic accuracy of those sounds is considered to be lower.

Auscultation of the respiratory system is noninvasive, safe, inexpensive, and easy to perform; therefore, it is a very common examination performed by many physicians [12,13]. However, a decline in physical examination skills has recently been highlighted [14], and 10%-24% of diagnostic errors are caused by misinterpretation or missed physical examinations [14,15]. The consequences of physical examination inadequacy include missed or delayed diagnosis in 76% of cases [14]. In particular, lung auscultation requires physicians to interpret sounds [13], and a meta-analysis reported that the sensitivity of lung auscultation is 37%, and the specificity is 89% [12]. In another study, the agreement between wheezes and crackles was low, with kappa values of approximately 50% and 40%, respectively [16]. The diagnostic accuracy of physicians substantially decreased when faced with discordant physical findings [15,17,18]; therefore, incorrect physical signs may lead to misdiagnosis. Although the absolute differences in diagnostic performance between single-ear and binaural auscultations in our study may appear modest, they were consistent across conditions and particularly pronounced for certain sound types, such as coarse crackles. These sounds are relevant in the diagnosis of pneumonia and interstitial lung disease. Therefore, even small reductions in auscultation accuracy may have meaningful clinical consequences, particularly in settings where timely and accurate bedside evaluation is essential.

Auscultation is a highly subjective process and depends on the physician’s technique [13]. Ambiguous identification and interpretation of sounds in auscultation can potentially lead to inaccurate diagnoses [13]. Previous studies have shown that pulmonologists have greater pulmonary auscultatory skills than interns in internal medicine, family practice doctors, and medical students [13,19]. However, in this study, even respiratory specialists could not improve the accuracy of single-ear auscultation. Furthermore, auscultation techniques are influenced by environmental noise, and in realistic clinical settings, the accuracy of auscultation might be lower than that in quiet environments [20]. In many hospitals, indoor environmental noise has been reported to exceed 50 dB [21]. Indeed, the environmental noise in the outpatient examination room in our hospital was similar. This study analysed auscultation in both quiet (<40 dB) and noisy environments (50-60 dB) and showed the low utility of single-ear auscultation in both settings. Although direct comparisons could not be performed because the sound questions used in this study were different, the results in both quiet and noisy environments were similar. This study revealed that single-ear auscultation is not recommended, even in quiet environments, and is certainly not recommended in outpatient examination rooms or emergency rooms.

The auscultation technique originated from the direct method, where physicians directly placed their ears on the patient’s chest [1]. The stethoscope was invented by René Theophile Hyacinthe Laënnec in 1816 and was originally made of a wooden tube used with a single ear [1,22]. With advances in technology, it evolved into the binaural stethoscope [22-24], designed to assess cardiovascular and respiratory sounds [20,25,26]​​​​​​​. Modern stethoscopes consist of a chest piece and binaural earpieces, with sound transmitted through hollow tubing to both ears [14,15,16]. We believe that this system not only increases the total amount of sound received but also helps seal the ears, reducing surrounding noise. Based on our research, physicians should use a binaural stethoscope correctly, listening with both ears.

This investigation has several limitations. The respiratory sounds used for the auscultation questions were recorded by a digital (electronic) stethoscope. There might be differences in sound between electronic and acoustic stethoscopes because electronic stethoscopes convert acoustic sound waves to electrical signals [26]. In our study, to avoid bias from differences between electronic and acoustic stethoscopes, all participants listened to a normal sound sample before examination. Additionally, electronic stethoscopes can be amplified and processed for optimal listening [26, 27], and filtering can enable electronic stethoscopes to mimic acoustic stethoscopes [27]. Furthermore, it was reported that electronic stethoscopes have superior sounds to acoustic stethoscopes [28,29]. Therefore, we believe that the difference between digital and acoustic stethoscopes is very small. The same sounds were used during both the first and second tests. Therefore, an interval of at least one week was set between the two tests. The majority of participants took the second test exactly seven days after the first, while a few completed it after slightly longer intervals due to scheduling constraints. No participants reported recognizing the reuse of the same, suggesting that the influence of memory effects was likely minimal. The order of single-ear and binaural auscultations might also introduce bias, as participants’ ears might become accustomed to listening with repeated auscultation, resulting in lower scores for auscultation questions for the auscultation performed first. However, participants listened to a normal sound sample before performing auscultation, and the order of single-ear and binaural auscultations was changed between the first and second tests. Binaural auscultations were performed first during the first test. Therefore, we believe that the bias in this study was minimal.

## Conclusions

This study demonstrated that the utility of single-ear auscultation for lung assessment was lower than that of binaural auscultation, with modest but consistent differences observed across environments and participant backgrounds. These differences were particularly notable for certain sounds, such as coarse crackles, which are relevant in diagnosing pulmonary diseases. Although clinical outcomes were not directly assessed, our findings suggest that single-ear auscultation may compromise diagnostic reliability. Physicians are therefore encouraged to use binaural auscultation, especially in settings where accurate respiratory evaluation is essential.
